# Therapeutic value of surgical paraaortic staging in locally advanced cervical cancer: a multicenter cohort analysis from the FRANCOGYN study group

**DOI:** 10.1186/s12967-018-1703-4

**Published:** 2018-11-26

**Authors:** Yohann Dabi, Vanille Simon, Xavier Carcopino, Sofiane Bendifallah, Lobna Ouldamer, Vincent Lavoue, Geoffroy Canlorbe, Emilie Raimond, Charles Coutant, Olivier Graesslin, Pierre Collinet, Alexandre Bricou, Emile Daraï, Cyrille Huchon, Marcos Ballester, Bassam Haddad, Cyril Touboul, For the Groupe de Recherche FRANCOGYN

**Affiliations:** 1 Department of Obstetrics and Gynecology, Centre Hospitalier Intercommunal, Faculté de médecine de Créteil UPEC – Paris XII, 40 Avenue de Verdun, 94000 Créteil, France; 2 Department of Obstetrics and Gynecology, Hopital Nord, APHM, Marseilles, France; 3 Department of Gynaecology and Obstetrics, Tenon University Hospital, Assistance Publique des Hôpitaux de Paris (AP-HP), Institut Universitaire de Cancérologie (IUC), University Pierre and Marie Curie, Paris 6, France; 4 Department of Obstetrics and Gynaecology, Centre hospitalier régional universitaire de Tours, hôpital Bretonneau, Tours, France; 5 Service de Gynécologie, CRLCC Eugène-Marquis, CHU de Rennes, Université de Rennes 1, Rennes, France; 6 Department of Gynaecology and Obstetrics, Pitié Salpetrière University Hospital, Assistance Publique des Hôpitaux de Paris (AP-HP), Institut Universitaire de Cancérologie (IUC), University Pierre and Marie Curie, Paris 6, France; 7 Department of Obstetrics and Gynaecology, Institute Alix de Champagne University Hospital, Reims, France; 8 Centre de lutte contre le cancer Georges François Leclerc, Dijon, France; 9 Department of Obstetrics and Gynecology, Centre Hospitalier Régional Universitaire, Lille, France; 10 Department of Obstetrics and Gynecology, Assistance Publique des Hôpitaux de Paris (AP-HP), Jean-Verdier University Hospital, Bondy, France; 11 Department of Gynaecology and Obstetrics, Intercommunal Hospital Centre of Poissy-Saint-Germain-en-Laye, 78103 Poissy, France

**Keywords:** Cervical cancer, Locally advanced cervical cancer, Nodal surgical staging, Paraaortic lymph nodes invasion

## Abstract

**Background:**

The prognostic impact of surgical paraaortic staging remains unclear in patients with locally advanced cervical cancer (LACC). The objective of our study was to evaluate the survival impact of surgical staging in patients with LACC and no evidence of paraaortic lymph node (PALN) metastasis on pre-operative imaging work-up.

**Methods:**

Data of 1447 patients with cervical cancer treated between 1996 and 2016 were extracted from maintained databases of 10 French University hospitals. Patients with locally advanced disease (IB2 or more) treated by concurrent chemoradiation therapy (CRT) and no evidence of paraaortic metastasis on pre-operative imaging work-up were selected for further analysis. The Kaplan–Meier method was used to estimate the survival distribution. A Cox proportional hazards model was used to account for the influence of multiple variables.

**Results:**

Six hundred and forty-seven patients were included, 377 (58.3%) with surgical staging and 270 (41.7%) without, with a mean follow up of 38.1 months (QI 13.0–56.0). Pathologic analysis revealed positive lymph nodes in 47 patients (12.5%). In multivariate model analysis, surgical staging remained an independent prognostic factor for DFS (OR 0.64, CI 95% 0.46–0.89, p = 0.008) and OS (OR 0.43, CI 95% 0.27–0.68, p < 0.001). The other significant parameter in multivariate analysis for both DFS and OS was treatment by intracavitary brachytherapy (OR respectively of 0.7 (0.5–1.0) and 0.6 (0.4–0.9), p < 0.05).

**Conclusion:**

Nodal surgical staging had an independent positive impact on survival in patients with LACC treated with CRT with no evidence of metastatic PALN on imaging.

**Electronic supplementary material:**

The online version of this article (10.1186/s12967-018-1703-4) contains supplementary material, which is available to authorized users.

## Background

Cervical cancer is the third most common cancer in women worldwide and around half of the patients are diagnosed with locally advanced cervical cancer (LACC) [1]. After multiple phase III studies demonstrated that concurrent chemoradiotherapy improved overall survival (OS) in patients with LACC, current guidelines recommend chemoradiation therapy (CRT) as the standard treatment for these patients [1, 2].

The latest FIGO classification for cervical cancer do not include lymph node status [3] despite considerable evidence reporting a major impact on prognosis [4, 5]. This may be because developing countries—where the incidence of cervical cancer is the highest—cannot afford imaging techniques such as positron emission tomography–computed tomography (PET-CT). However, determining paraaortic lymph node (PALN) status would appear to be of paramount importance to tailor adjuvant concurrent chemoradiation therapy (CRT) and personalize the fields of radiation [6, 7]. Personalized radiation fields are mandatory to prevent unnecessary radiation and the associated morbidity.

The debate about the most effective way to assess PALN status is ongoing. On one hand, imaging exams are non-invasive but lacks sensitivity for detecting PALN metastasis especially in cases of micrometastases [8]. On the other hand, surgical staging is invasive but is associated with a low rate of complications in well-trained teams [9] and provides robust results for PALN evaluation. The prognostic impact of surgical paraaortic staging remains unclear in patients with LACC and there are some discrepancies in the scientific literature regarding this issue [10–12]. The benefit of correctly identifying a higher proportion of patients with PALN by surgical staging could be tempered by a delay in initiating CRT and surgical morbidity [11]. It is thus important to determine whether surgical staging has any impact on survival and disease recurrence.

The objective of our study was to evaluate the survival impact of paraaortic nodal surgical staging in patients with LACC and no evidence of PALN metastasis on pre-operative imaging work-up.

## Methods

We conducted a retrospective study using maintained databases from 10 French institutions (Creteil University Hospital, Tenon University Hospital, Poissy University Hospital, Reims University Hospital, Lille University Hospital, Tours University Hospital, Bondy University Hospital, Rennes University Hospital, and Marseille Public Hospital North). These databases registered all patients diagnosed with cervical cancer at any stage between January 1996 and December 2016. The research protocol was approved by the Institutional Review Board (IRB) of the French College of Obstetrics and Gynaecology (CEROG 2016-GYN-0502).

Patients with locally advanced cervical cancer (LACC) treated with CRT and no distant or para-aortic invaded nodes on pre-treatment computed tomography scanner (CT-scan) or PET-CT were selected for further analysis. LACC was defined as patients with at least stage IB2 according to the latest 2009 International Federation of Gynecology and Obstetrics (FIGO) classification. Exclusion criteria were: patients with stage IVB; patients treated by radiotherapy only; and patients with missing data for surgical staging.

The decision to perform surgical paraaortic staging or other complementary therapies (extended field radiotherapy, intracavitary brachytherapy, completion surgery) was center–driven. As for all aspects of patient management, the decision was made within a multidisciplinary committee and was based on both patient and tumor characteristics. During surgical para-aortic staging, all of the lymphatic tissue from the aorta was removed from the iliac bifurcation to the left renal vein. Pelvic lymphadenectomy was not routinely performed since the area is covered by traditional pelvic radiation fields. All patients were subsequently treated by CRT and received pelvic conformational radiotherapy at the total dose of 45 Grays (25 fractions) in 5 weeks with a concomitant 40 mg/m^2^ weekly base of cisplatinium ± 5FU in some centers. Some patients received intracavitary brachytherapy (15 grays) to complete pelvic conformational radiotherapy.

Patients with positive nodes after surgical staging were supposed to receive an extension of the radiation fields in the paraaortic region. However, some patients without surgical para-aortic staging also received paraaortic radiation therapy in the case of pelvic lymph node involvement on PET-CT. Some centers commonly performed completion surgery (hysterectomy) following RCT in patients with residual disease.

Follow-up protocol included a gynecologic examination every 3 months for 2 years and then every 6 months for 2 years. Magnetic resonance imaging (MRI) or a PET-CT scan were performed when clinically indicated. Recurrences were diagnosed either on biopsy or with an imaging exam.

According to previous reports, we applied the following definitions to stratify the sites of recurrence: (i) local recurrence was defined as a vaginal or central pelvic location without lymph node involvement; (ii) regional recurrence was defined as a non-central pelvic location or a peritoneal carcinomatosis and no lymph node involvement; (iii) nodal recurrence included pelvic and/or paraaortic nodal locations; (iv) distant recurrence included distant metastasis (bone, liver, lung and brain); (v) multiple site recurrence included any combination of the locations mentioned above.

The date of the end of primary treatment was used to calculate disease free survival (DFS) and OS.

Databases were managed using Excel (Microsoft Corporation, Redmond, WA, USA) and statistical analyses were performed using R software (3.3.1 version, available online). Statistical analysis was based on the Student’s t test for continuous variable and the χ^2^ test or Fisher’s exact test for categorical variables. The Kaplan–Meier method was used to estimate the survival distribution. Comparisons of survival were made using the log rank test. A Cox proportional hazards model including all the parameters statistically significant in univariate analysis, was used to account for the influence of multiple variables. Values of p < 0.05 were considered to denote significant differences.

## Results

**Fig. 1:**
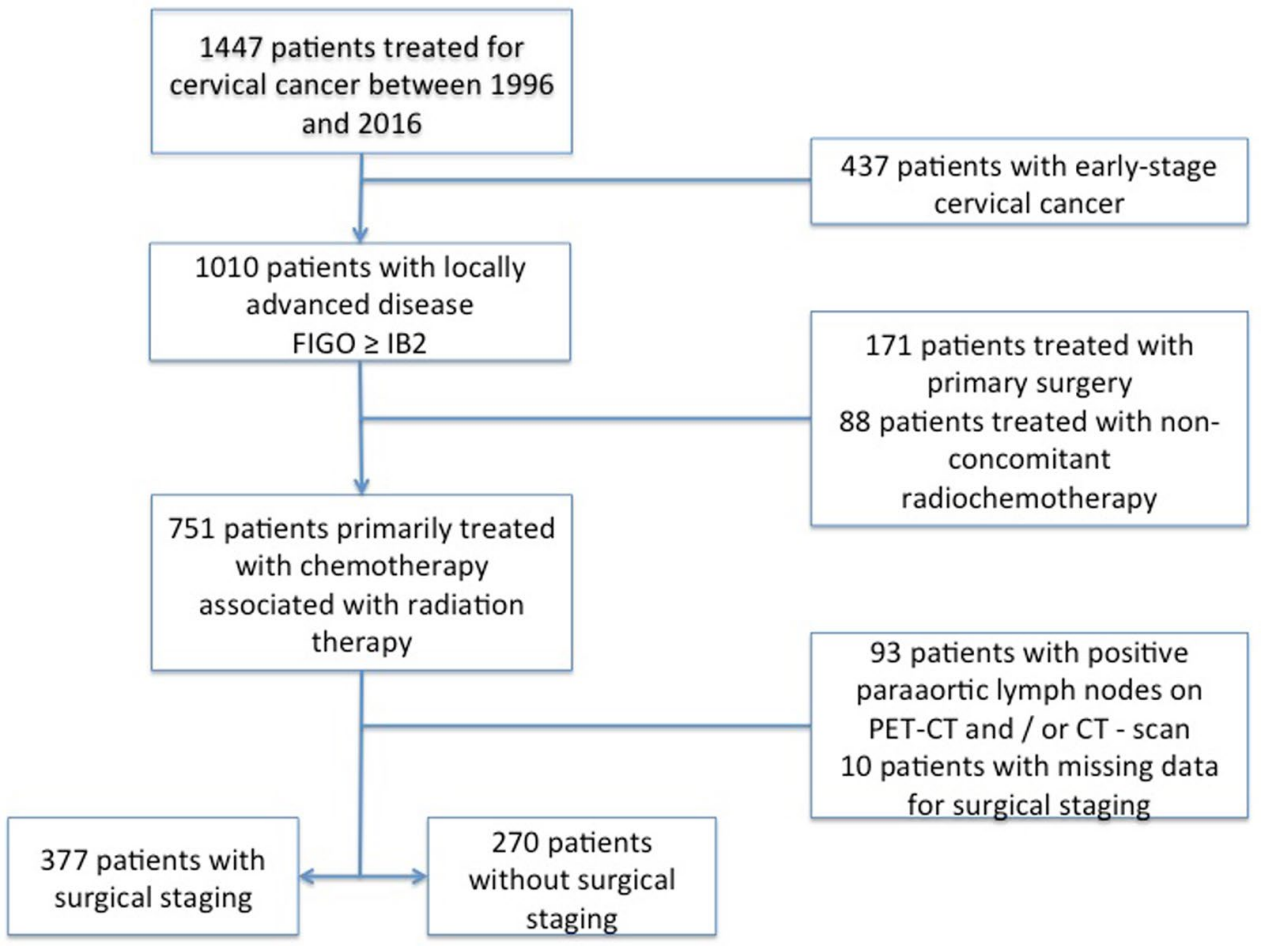
Flow chart of the study

Between 1996 and 2016, 1447 patients were treated for cervical cancer within our institutions. Among them, 647 fulfilled the inclusion criteria and were included for analysis: of these, 377 had undergone surgical staging and 270 had not (Fig. 1).

The main characteristics of the patients included are presented in Table 1. Two hundred and seventy-six patients (42.7%) had a pre operative CT-scan to assess lymph nodes status and 371 (57.3%) a PET-CT. Most patients had a tumor > 4 cm and 53% received intracavitary brachytherapy. Surgical staging was laparoscopic in all but five patients (2 laparotomy, 1 robot assisted, 2 laparoscopy converted during procedure to open laparotomy). Eighteen patients (4.8%) experienced per-operative complications (mostly vascular) and 50 (13.3%) postoperative complications of any severity. Among the patients with surgical staging, 47 (12.5%) had positive paraaortic lymph nodes on final pathologic analysis.

**Table 1:** Main characteristics of the patients included

	N = 647
Age (years)	54.4 (44–64)
BMI	25.7 (21.1–29.4)
Hormonal status^a^	
Menopausal	355 (54.9)
Childbearing	282 (43.6)
Parity	2.7 (1–3)
Pathologic type	
Squamous cell	530 (81.9)
Adenocarcionma	88 (13.6)
Other	29 (4.5)
FIGO stage^a^	
IB2	86 (13.3)
IIA	58 (9.0)
IIB	359 (55.5)
III	69 (10.7)
IV	66 (10.2)
Pre-operative imaging	
CT-scan	276 (42.7)
PET-CT	371 (57.3)
Tumor size on MRI	
< 40 mm	194 (30.0)
40–60 mm	278 (43.0)
> 60 mm	112 (17.3)
Unknown	63 (9.7)
Concomitant chemoradiation (CRT)	647 (100)
Intracavitary brachytherapy	343 (53.0)
RCC Boost in paraaortic lymph nodes	153 (23.6)
Completion surgery after CRT	290 (44.8)

Data are expressed either as mean (interquartile range) or as n (%)

^a^ Missing data for 10 patients (hormonal status) and 9 patients (stage)

### Survival analysis

**Fig. 2:**
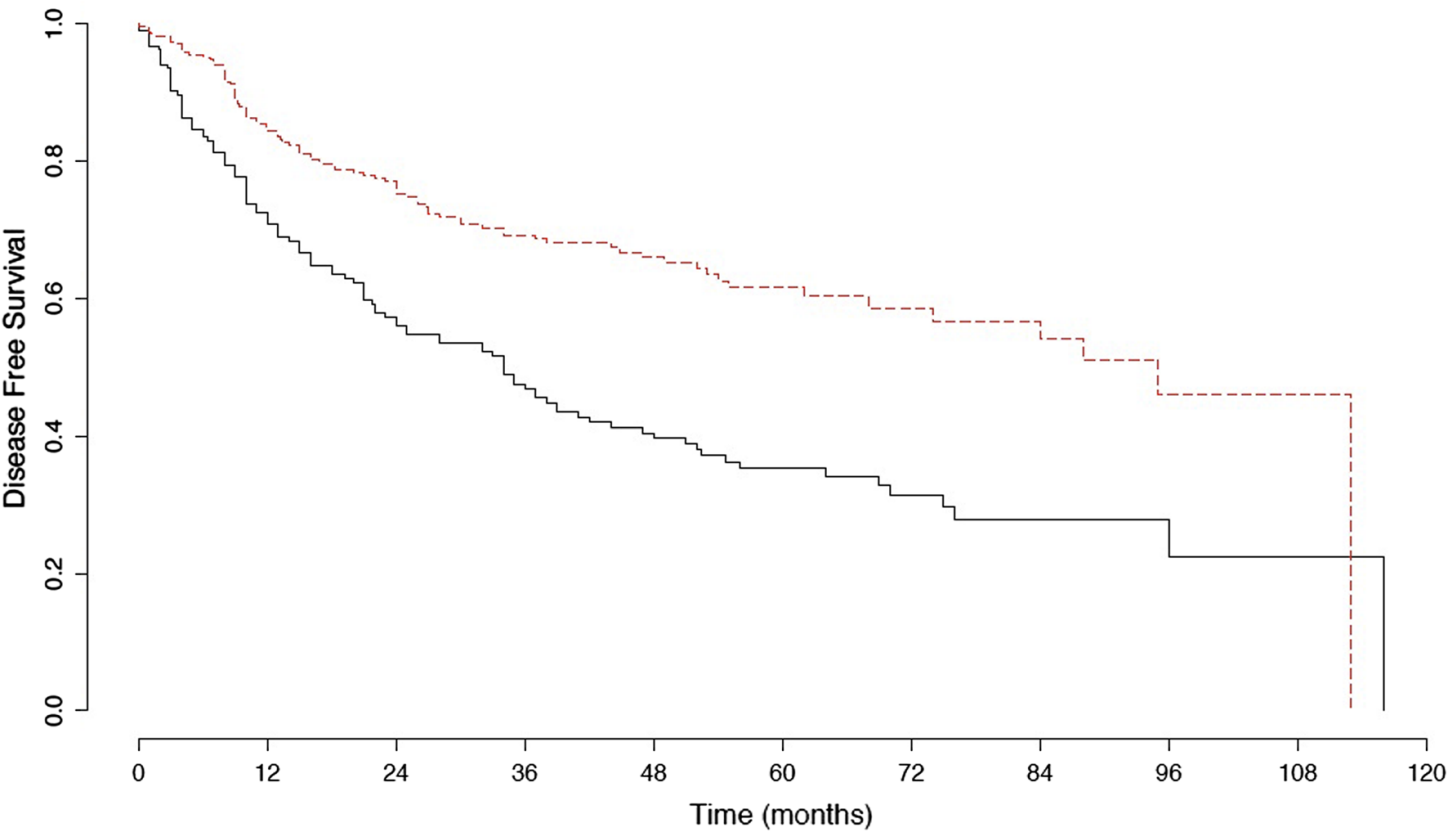
Kaplan Meier curve for disease free survival in patients with and without surgical staging. Red dashed line: patients with surgical staging. Black dashed line: patients without surgical staging. The difference was statistically different between the two groups (p < 0.001)

**Fig. 3:**
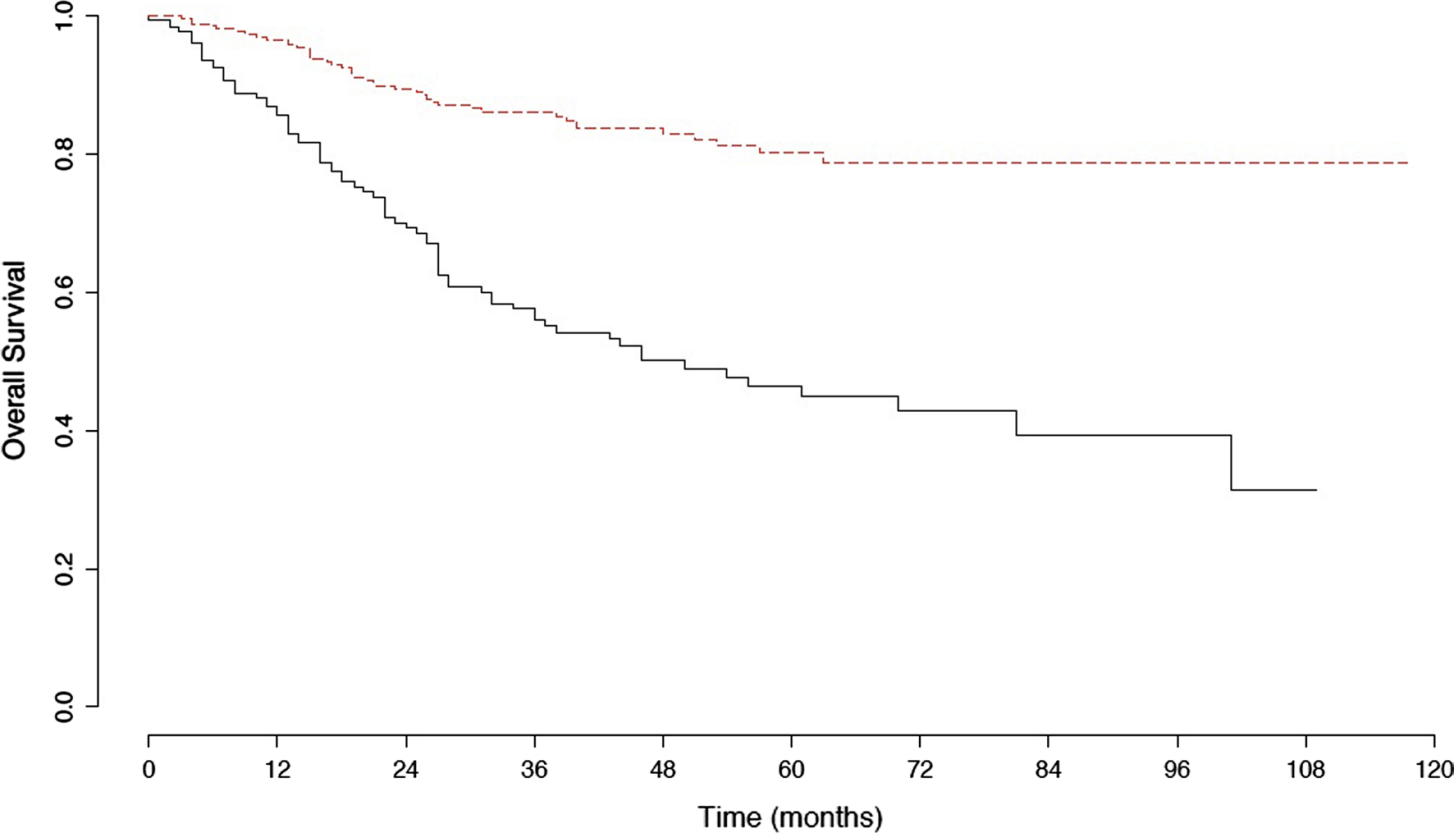
Kaplan Meier curve for overall survival in patients with and without surgical staging. Red dashed line: patients with surgical staging. Black dashed line: patients without surgical staging. The difference was statistically different between the two groups (p < 0.001)

Mean follow up was 38.1 months (QI 13.0–56.0). During follow up, 140 patients died: 53 patients (14.1%) with surgical staging and 87 (32.2%) without. Two hundred and two patients experienced recurrance during follow up: 102 with surgical staging and 100 without. Surgical staging was significantly associated with better DFS and OS than clinical staging (p < 0.001) (Figs. 2 and 3). Para-aortic radiotherapy boost was not associated with a difference in survival. Patterns of recurrence are presented in Table 2. There were no differences in the site of recurrence between patients with and without surgical staging. Most patients had either local, distant or multiple site metastases. Within patients that had surgical paraaortic lymph nodes staging, patients with histologically confirmed lymph nodes metastases had significantly worse overall survival than those with no evidence of metastases on final pathological analysis (p < 0.01) (Additional file 1: Figure S1).

**Table 2:** Patterns of recurrence in patients with and without surgical staging

	With staging 102 patients (%)	Without staging 100 patients (%)	p-value
Local	30 (29.4)	22 (22)	0.72
Regional	8 (7.8)	6 (6)	
Distant	26 (25.5)	26 (26)	
Lymph node	8 (7.8)	9 (9)	
Multiple sites	30 (29.4)	36 (36)	

Missing data: one patient without staging

### Multivariate model analysis

Results of the multivariate model analysis for factors influencing DFS and OS are presented in Table 3. Surgical staging remained an independent prognostic factor for DFS (OR 0.64, CI 95% 0.46–0.89, p = 0.008) and OS (OR 0.43, CI 95% 0.27–0.68, p < 0.001) in multivariate analysis. The other parameter that remained significant for both DFS and OS was treatment by intracavitary brachytherapy associated with CRT (respectively OR 0.7 (0.5–1.0) p = 0.04 and 0.6 (0.4–0.9) p = 0.02). This parameter was not different in the two study groups (p = 0.24).

**Table 3:** Multivariate analysis of factors influencing DFS and OS using cox model

Variable	DFS			OS		
	OR	CI (95%)	p-value	OR	CI (95%)	p-value
Age at diagnosis	1.0	1.0–1.0	0.69	1.0	1.0–1.0	0.98
BMI ≥ 30	1.1	0.7–1.6	0.68	1.1	0.7–1.8	0.7
FIGO stage ≥ III	2.2	0.9–4.9	0.06	3.8	1.5–9.8	*0.006*
Tumor size ≥ 4 cm	1.1	0.8–1.6	0.52	0.8	0.5–1.4	0.49
Hydronephrosis on pre-RCC MRI	0.8	0.5–1.4	0.44	0.7	0.4–1.3	0.31
Parametrial invasion on pre-RCC MRI	1.0	0.5–1.7	0.89	0.5	0.3–1.0	0.057
Surgical paraaortic staging	0.64	0.46–0.89	*0.008*	0.43	0.27–0.68	*< 0.001*
Intracavitary brachytherapy	0.7	0.5–1.0	*0.04*	0.6	0.4–0.9	*0.02*
Completion surgery following CRT	0.7	0.5–1.0	*0.03*	1.0	0.6–1.6	0.91

Significant factors for both DFS and OS are surgical staging and intracavitary brachytherapy associated with RCC

## Discussion

Our study shows that surgical paraaortic staging is associated with increased OS and DFS in patients with LACC treated with CRT and with no evidence of PALN metastasis on pre operative imaging.

We believe that two parameters are responsible for fueling the debate around this controversy that has lasted for many years. The first is the confusing role of the pre-operative imaging in the initial assessment of patients with LACC, and the second is the role of extended radiation fields in these patients.

As mentioned in the introduction, current FIGO classification is based on clinical staging. However, in developed countries, most patients have a CT-scan or a PET-CT pre-operatively to assess initial disease spread with high true positive value for identification of positive lymph nodes, especially for PET-CT [13]. When PET-CT shows an uptake in the paraaortic area, extended radiation fields should be applied and surgical staging would seem to be at best unnecessary and at worst harmful. False negative rates for PET-CT in the paraaortic area have been reported to be as high as 13% in patients with LACC [12, 14–17] with a low sensitivity of detection of small node disease: 22% if histologically confirmed PA nodal metastasis < 5 mm in size [15] as well as failure to identify most patients with peritoneal disease. This underlines the lack of sensitivity of PET-CT for small volume metastases in PALN. Our inclusion criteria resulted in selecting patients either without metastases or with small volume metastases only. In this population, surgical staging would increase occult metastasis detection. Increased DFS and OS in patients with surgical staging clearly demonstrate the therapeutic effect of PALN dissection. In our cohort, 47 patients (12.5%) had positive PALN on final pathologic analysis and these patients, with small volume metastases, probably benefited the most from the surgical staging.

In our cohort, only a small proportion of patients had an extended radiation field in the paraaortic area (23.6%) and this proportion was similar in patients with and without surgical staging. The decision of whether to apply extended radiation fields was thus not based on the results of the surgical staging. As mentioned by Pomel et al. [18], no study has shown a clear benefit of extended field radiotherapy on survival following the introduction of cisplatin systemic therapy in the initial management of patients with LACC [19]. The benefit of surgical staging in patients with negative preoperative workup seems to be independent of the extent of the radiation fields. Moreover, patterns of recurrence in patients with or without surgical staging are similar, with most recurrences occurring locally or at distant sites. Such patterns emphasize the need to improve local tumor-control in patients with LACC.

We recognize that the retrospective nature of our study limits the generalization of our findings. However, this cohort is the largest ever reported and large multicenter cohorts are of utmost importance to accumulate evidence to resolve this long-standing controversy. Patients in our cohort had either a CT-scan or a PET-CT pre operatively despite the fact PET-CT is known a higher sensitivity for detecting lymph nodes metastases. Our choice to also include patients with CT-scan was driven by the fact that most centers do not have routinely access to PET-CT pre-operatively. Because of the retrospective nature of our study, data regarding recurrences’ management was not available. As we report the therapeutic benefit of surgical paraaortic dissection prior to initiation of concomitant radio-chemotherapy, salvage paraaortic lymph nodes removal in patients experiencing lymph nodal recurrence using minimally invasive surgery could be a valid therapeutic approach as recently suggested by Gallotta et al. [20].

A commonly used argument against surgical staging is the subsequent delay in starting concomitant chemo-radiotherapy. We were not able to evaluate this parameter but in light of our results, with increased survival rates in patients with surgical staging, it is safe to think that this hypothesis can be ruled out. This is all the more true when taking into consideration that others have reported no significant delays in starting chemoradiation therapy in these patients [21]. Finally, while surgical morbidity in our cohort was acceptable, most of the participating centers have a considerable expertise in laparoscopic staging in gynecologic malignancies. Generalization of surgical staging to centers with less experience might result in greater morbidity with a negative impact on survival. Recently, some authors developed nomograms to predict paraaortic lymph nodes invasion in patients with locally advanced cervical cancer. As this approach might be of interest, these nomograms usually lack of sensitivity and are not validated in prospective cohort yet [22].

The complex interactions between the different variables determining prognosis, have delayed initiation of a randomized controlled trial to answer the issue. The LILACS study by Frumovitz et al. [23] should bring interesting results and provide us with some answers. To date, the only randomized trial, conducted by Lai et al. [11], concluded that clinical staging led to better DFS and OS than surgical staging. However, this study has been highly criticized with major differences in patient characteristics between the groups and more patients receiving concurrent chemotherapy in the radiologically staged group compared with the surgically staged group. The trial was ended prematurely without reaching its primary endpoint. On the other hand, some retrospective studies have suggested a positive survival impact of surgical staging [10, 24]. Our study is in line with these and, by virtue of including more patients than the previous studies, could serve as a basis to design further prospective trials.

## Conclusion

We found surgical staging had a therapeutic value in women with node metastases not detected on pre-operative imaging, with significant improvement in DFS and OS achieved by tailoring radiation therapy plans or modifying planned therapy, and identifying patients with peritoneal spread. This benefit could vary from one patient to another due to the numerous therapeutic factors involved in improving survival, as well as from one center to another as experience in laparoscopic staging is a determining factor to limit associated morbidity. Further studies should help select patients that will benefit the most from surgical staging.

## Additional file

**Additional file 1: Figure S1.** Kaplan–Meier curve for overall survival in patients with surgical staging stratified by final pathological analysis of paraaortic lymph nodes. In black: patients without lymph nodes metastases. In red: patients with paraaortic lymph nodes metastases.
