# Supplementary figures and images for: Therapeutic value of surgical paraaortic staging in locally advanced cervical cancer: a multicenter cohort analysis from the FRANCOGYN study group
Source: J Transl Med. 2018 Nov 26;16:326. doi: 10.1186/s12967-018-1703-4 (PMC6260775; doi:10.1186/s12967-018-1703-4)

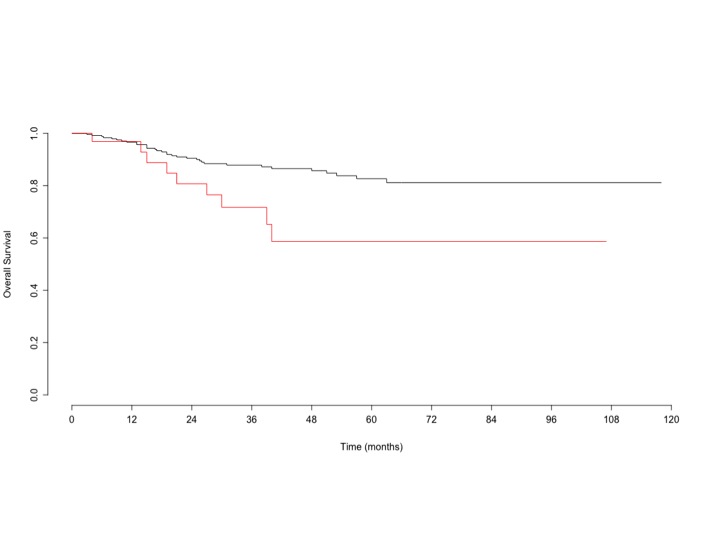

Supplement: Supplementary file 1 — Additional file 1: Figure S1. Kaplan–Meier curve for overall survival in patients with surgical staging stratified by final pathological analysis of paraaortic lymph nodes. In black: patients without lymph nodes metastases. In red: patients with paraaortic lymph nodes metastases. [file 12967_2018_1703_MOESM1_ESM.jpg]
